# Spectroscopic studies on the interaction between erlotinib hydrochloride and bovine serum albumin

**Published:** 2010

**Authors:** F. Rasoulzadeh, D. Asgari, A. Naseri, M.R. Rashidi

**Affiliations:** 1 Department of Chemistry, Faculty of Science, Islamic Azad University; 2 Drug Applied Research Center, Tabriz University of Medical Sciences; 3 Department of Analytical Chemistry, Faculty of Chemistry, University of Tabriz, Tabriz, Iran

**Keywords:** Erlotinib hydrochloride, Albumin, Fluorescence quenching

## Abstract

**Background and the purpose of the study:**

The binding ability of a drug to serum albumin has influence on the pharmacokinetics of a drug. In the present study, the mutual interaction of anticancer drug erlotinib hydrochloride with bovine serum albumin (BSA) using fluorescence and UV/vis spectroscopy was investigated.

**Methods:**

The BSA solution (0.1 mM) was prepared daily in Tris buffer (0.05 mol l-1, pH =7.4) and treated at final concentration of 1.67×10-5 M with different amounts of erlotinib hydrochloride to obtain final concentrations of 0, 0.2, 0.4, 0.8, 1, 2, 4, 6, 8, 20 and 42 µM receptively. The mixture was allowed to stand for 5 min and the fluorescence quenching spectra were recorded at 298, 303, 308 and 313 K.

**Results:**

It was found that erlotinib hydrochloride caused the fluorescence quenching of BSA by the formation of a BSA-erlotinib hydrochloride complex. The mechanism of the complex formation was then analyzed by determination of the number of binding sites, apparent binding constant *K*, and calculation of the corresponding thermodynamic parameters such as the free energy (△G), enthalpy (△H) and entropy changes (△S) at different temperatures. Results showed that binding of erlotinib hydrochloride to BSA was spontaneous, and the hydrophobic forces played a major role in the complex formation. The distance, *r*, between donor (BSA) and acceptor (erlotinib hydrochloride) was found to be less than 8 nm suggesting the occurrence of non-radiative energy transferring and static quenching between these two molecules.

**Conclusion:**

The results provided preliminary information on the binding of erlotinib hydrochloride to BSA and the presence of a single binding site on BSA and *K* values for the association of BSA with erlotinib hydrochloride increased by the increase in temperature.

## INTRODUCTION

**Figure 1:**
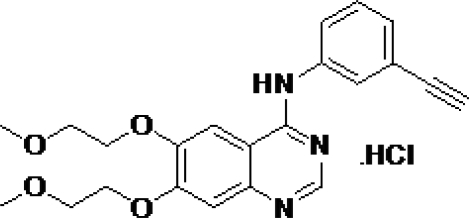
Erlotinib hydrochloride.

Erlotinib hydrochloride (Figure 1) belongs to a group of anti cancer drugs known as inhibitors of epidermal growth factor receptor (EGFR) tyrosine kinase which is highly expressed in different forms of cancers. This drug binds to ATP- binding site in a reversible manner and disables phosphorylating ability of the oncogenic EGFR and inhibits signal transduction cascade causing apoptosis of malignant cells. Erlotinib hydrochloride is used for treatment of non-small cell lung cancer, pancreatic, ovarian, head and neck cancers^1^. Plasma protein binding of erlotinib hydrochloride in mouse, rat, and human has been evaluated to be 95, 92, and 92%, respectively (2).

Serum albumins are the major soluble protein constituents of the circulatory system possessing many physiological functions of which the most important are serving as a depot and a transport protein for many endogenous and exogenous compounds such as drugs (3). Bovine serum albumin (BSA), due to its structural similarity to human serum albumin (HSA) and considerable stability, has been used to replace human serum albumin in protein- drug studies (2). BSA consists of three homologous domains (I, II, III) and each domain in turn is the product of two sub-domains (4). BSA has two tryptophans residues, Trp-134 and Trp-212, which are embedded in the first sub-domain IB and sub- domain IIA, respectively (3). The high sensitivity of tryptophan residues to its local environment produces valuable intrinsic fluorescence properties in BSA molecule. Changes in emission spectra of tryptophan are common in response to protein conformational transitions, subunit association, denaturation or substrate binding.

Since the binding ability of a drug to serum albumin may have an important impact on pharmacokinetics as well as the determination of the dosage form of the drug (5), changes in the intrinsic fluorescence intensities of BSA-drug complex could provide considerable information regarding the binding characteristics and the therapeutic effectiveness of drugs (6).

Although, the fluorescence quenching study of BSA interactions with many compounds including drugs using fluorescence spectroscopy have been thoroughly investigated and reported (4), to the best of our knowledge, the binding profile of erlotinib hydrochloride to this class of proteins has never been investigated. In this study, the properties of binding between erlotinib hydrochloride and BSA were investigated using fluorescence quenching method and UV/vis absorption spectroscopy. The aim of this study was to analyze the fluorescence quenching mechanism of BSA by erlotinib hydrochloride, the number of the biding sites, the specific binding pocket, and the effects of erlotinib hydrochloride on the conformational changes of BSA.

## MATERIAL AND METHODS

### 

#### Material

Erlotinib hydrochloride was synthesized as described before (7). BSA was purchased from Sigma-Aldrich (Dorset, UK). All other reagents were obtained from Merck (Darmstadt, Germany).

#### Spectral measurements

The UV spectrum was recorded at room temperature on a Shimadzu 2550 UV/VIS Spectrophotometer (Shimadzu, Japan) equipped with 3.0 cm quartz cells. All fluorescence spectra were recorded on RF-5301 Spectrofluorimeter (Shimadzu, Japan) equipped with a xenon lamp source, a 1.0 cm quartz cell and a thermostat bath. The widths of both excitation and emission slits were set to 5 nm. The optimum excitation and emission wavelengths for BSA were found to be 295 and 339 nm, respectively. The resulting fluorescence data were corrected for the background fluorescence of buffer and erlotinib hydrochloride.

#### Procedures

The stock solution of erlotinib hydrochloride (0.25 mM) was prepared in Tris buffer solution (0.05 mol l-1, 0.1 mol l-1 NaCl, pH=7.4). The BSA solution (0.1 mM) was prepared daily in Tris buffer (0.05 mol l-1, pH =7.4) and treated at final amounts of 1.67×10-5 M with different concentrations of erlotinib solution to give concentrations of 0, 0.2, 0.4, 0.8, 1, 2, 4, 6, 8, 20 and 42 µM respectively. The mixture was allowed to stand for 5 min and the fluorescence quenching spectra were recorded at 298, 303, 308 and 313 K.

## RESULTS AND DISCUSSION

### 

#### Fluorescence quenching spectra

The fluorescence intensity of a compound is decreased by a variety of molecular interactions such as excited-state reactions, molecular rearrangements, energy transfer, static, and dynamic quenching (8). Such decrease in intensity is called fluorescence quenching. Static quenching refers to formation of complex between quencher and the fluorophore, while dynamic quenching refers to the collision of the quencher and fluorophore during the excitation process. Using Stern-Volmer equation (Eq. [1]) and analyzing the fluorescence quenching data, the fluorescence quenching relationship may be predicted as:

1 $$F_{0}/F=1+K_{q}\tau_{0}[\text{Q}]=K_{sv}[\text{Q}]$$

where *F_0_* and *F* are the fluorescence intensities before and after addition of the quencher, respectively, *K*, and [Q] are the quenching rate constant of the bimolecular, the Stern-Volmer dynamic quenching constant, the average lifetime of the bimolecular without quencher and the concentration of the quencher, respectively. Equation [1] was applied to determine *K* by linear regression of a plot of *F_0_/F* versus [Q].

**Figure 2:**
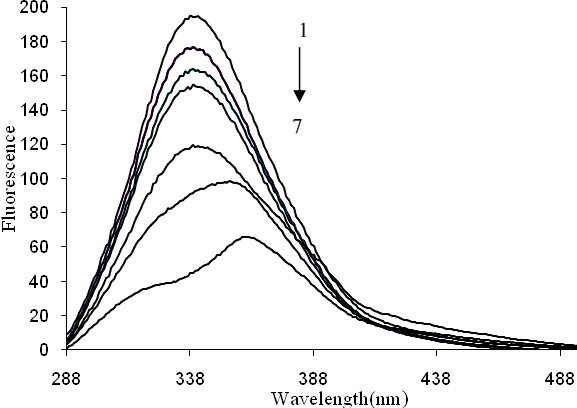
Fluorescence spectra of BSA in the presence of various concentrations of erlotinib hydrochloride in Tris buffer (0.05 mol l^-1^, pH =7.4) at 313 K (λ=339 nm). BSA concentration: 1.67×10^-5^ M, the concentration of erlotinib hydrochloride (1→7): 0, 0.6, 4, 6, 8, 20 and 42 µM.

Figure 2 shows changes in the fluorescence intensity by addition of erlotinib hydrochloride at different concentrations to BSA solutions. As it is seen, presence of erlotinib hydrochloride in BSA solution, even at low concentrations, resulted in fluorescence quenching of the BSA molecule, and the amount of fluorescence quenching was dependent on the concentration of erlotinib hydrochloride molecules in the BSA solution. At higher erlotinib hydrochloride concentrations, a slight blue shift was produced indicating intermolecular binding between erlotinib hydrochloride and BSA.

In order to obtain the results within the linear concentration of erlotinib hydrochloride, the curves have linear relationships, and the slopes increases by the increase in temperature, thereby indicating the occurrence of a dynamic quenching interaction between erlotinib hydrochloride and BSA. Moreover, in dynamic quenching, diffusion plays an important constants are expected to increase by the increase in temperature. In table 1, the binding constants obtained by the Stern-Volmer method for erlotinib hydrochloride-BSA complex are listed.

**Table 1:** Stern Volmer quenching constant of the systems of Erlotinib hydrochloride-BSA at different temperatures.

*pH*	*T(K)*	*K_sv_(Lmol^−1^) ×10^−4^*	*K_q_(Lmol^−1^s^−1^)×10^−12^*	*R^a^*	*Regression equation*	*SD^b^*
7.4	298	3.6046	3.6046	0.9810	Y=0.0516x+0.9394	0.0125
	303	3.8272	3.8272	0.9840	Y=0.0542x+0.9453	0.0158
	308	3.9144	3.9144	0.9928	Y=0.0629x+0.9453	0.0201
	313	4.5931	4.5931	0.9940	Y=0.0752x+0.9805	0.0234

a *R* is the correlation coefficient.

b SD is standard deviation.

In a collisional or dynamic quenching the fluorophore and the quencher contact each other during the lifetime of the excited state, whereas in a static quenching a complex is formed between the fluorophore and the quencher. It is possible to distinguish static and dynamic quenching through the study of their dependency to temperature and viscosity, or by lifetime measurements. Generally, the collisional quenching constant of various kinds of quenchers with biomolecule is 2.0×1010 l mol-1s-1. However, the rate constant of the protein quenching initiated by erlotinib hydrochloride was found to be much greater than the maximum collision quenching constant of biomolecule, indicating that the quenching process is static. In addition, ground state complex by absorption spectra also indicates a static quenching involvement. The dynamic quenching only affects the excited state of quenching molecule with no function on the absorption spectrum of quenching substances.

#### Binding constant and binding sites

The apparent binding constant *K* and binding sites *n* for a small molecule that binds independently to a set of equivalent sites on a macromolecule (9) can be obtained from the following equation.

2 $$\log\left(F_{0}-F\right)/F=n\log K_{A}-n\log\left(\frac{1}{([Q]-\left(F_{0}-F)[P\right]/F_{0})}\right)$$

where *F_0_* and *F* are the fluorescence intensities before and after the addition of the quencher, [*Q*] and [P] are the total concentrations of quencher and protein, respectively. By plotting log (*F_0_* -*F*)/*F* versus log (1/ role and since higher temperatures result in larger ([*Q*] - (*F_0_* – *F*) [*P*]/*F_0_*)), the number of binding sites, diffusion coefficients, the bimolecular quenching *n*, and binding constant *K_a_* can be obtained.

**Figure 3:**
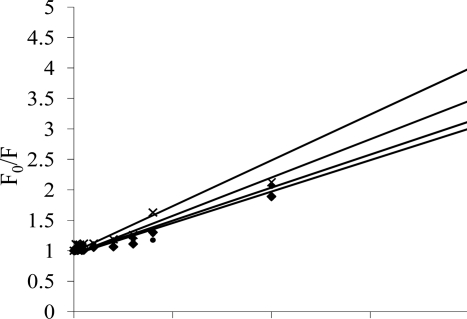
The Stern-Volmer plot for the quenching of BSA by erlotinib hydrochloride at 298°K(■), 303°K(♦), 308°K(•) and 313°K (×). pH 7.40, λ_ex_=295 nm and λ_em_=339 nm.

In the table 2, the binding constants, *K_0_*, and binding sites, n, for erlotinib hydrochloride associated with BSA are listed. The correlation coefficients are larger than 0.98 indicating that the interaction between erlotinib hydrochloride and BSA is well in agreement with the site-binding model underlined in Eq. [2]. The *K* values for association of erlotinib hydrochloride with BSA increased by the rise in temperature which may indicate the formation of a stable complex at higher temperatures (10) and it is consistent with the dynamic quenching mechanism obtained for the interaction of erlotinib with BSA. Dynamic quenching which depends on collisions between the excited state and the quencher is a diffusion-controlled process, and increases with temperatures. The obtained values for *n* were found to be 1 indicating that only a single binding site exists in BSA for erlotinib hydrochloride molecules. This number is in agreement with the reported numbers (5) Figure 3

**Table 2:** binding constant, *K*, the number of binding sites, *n*, and the thermodynamic parameters for the association of Erlotinib hydrochloride with BSA.

*pH*	*T(K)*	*K_a_(1mol^−1^)*×*10^−4^*	*n*	*△H (kJ mol^−1^)*	*△G (kJ mol^−1^)*	*△S (J mol^−1^)*
7.4	298	2.9882	1.1165	38.910	−25.838	217.278
	303	5.4250	1.2357		−26.924	
	308	5.6809	1.0457		−28.011	
	313	6.9838	0.9692		−29.097	

#### Thermodynamic Parameters and Nature of Binding

There are essentially four types of non-covalent interactions that play a key role in binding ligand to proteins.

These are hydrogen bonds, Van der Waals forces, electrostatic and hydrophobic bonds interactions (11–13). The thermodynamic parameters dependency to temperature must be obtained in order to elucidate the interaction forces between erlotinib hydrochloride and BSA. If the enthalpy change (△*H*) over the temperature range under study is minimal, then, the thermodynamic parameters △*S,* and △*G* can be determined by the Van't Hoff equation (Eq.[3-5]) (12):

3 $$\text{In}({\text{K}}_{\text{2}}/{\text{K}}_{\text{1}})=\Delta \text{H}/\text{R}(\text{1}/{\text{T}}_{\text{1}}-\text{1}/{\text{T}}_{\text{2}})$$

4 $$\Delta \text{G}=-\text{RT In}{\text{K}}_{\text{a}}$$

5 ΔG=ΔH-TΔS

where *K* is the binding constant and *R* is the gas constant.

Enthalpy change (△*H*), entropy change (△*S*) and free energy change (△*G*) for the binding interaction between erlotinib hydrochloride and BSA were calculated using Eq. [3–5] (9, 11–13). The thermodynamic parameters for the interaction of erlotinib hydrochloride with BSA are shown in table 2. The negative values of △*G* indicate that the binding process is spontaneous. The enthalpy (△*H*) and entropy (△*S*) of the interaction of erlotinib hydrochloride and BSA are positive. According to the report of Ross and Subramanian (12), the positive △H and △S value is associated with hydrophobic interaction, the negative △H and △S values are associated with hydrogen bonding and Van der Waals interaction in low dielectric medium. Finally very low positive or negative △H and positive △S values are characterized by electrostatic interactions. Thus, it is difficult to interpret the thermodynamic parameters of BSA- erlotinib interaction with a single intermolecular force. Therefore, the binding of erlotinib to BSA might involve strong hydrophobic interaction as evidenced by the positive values of △S while electrostatic interaction can not be excluded.

#### Fluorescence resonance energy transfer (FRET) from BSA to erlotinib hydrochloride

Fluorescence resonance energy transfer (FRET) is a reliable method for studying protein-ligand interactions and evaluation of the distance between the ligand and tryptophan residues of the protein.

The energy transfer efficiency E is defined by the following Eq. [6], where r is the distance from the ligand to the tryptophan residue of the protein, and is the Forster critical distance, at which 50% of the excitation energy is transferred to the acceptor. R can be calculated from donor emission and acceptor absorption spectra using the Forster formula Eq. [7].

6 $$\text{E}=\text{1}-\frac{\text{F}}{{\text{F}}_{\text{0}}}=\frac{{\text{R}}_{\text{0}}^{\text{6}}}{{\text{R}}_{\text{0}}^{\text{6}}+{\text{r}}_{\text{0}}^{\text{6}}}$$

7 $${\text{R}}_{\text{0}}^{\text{6}}=\text{8.79}\times \text{1}{\text{0}}^{-\text{25}}{\text{K}}^{\text{2}}{\text{N}}^{-\text{4}}\varphi \text{J}$$

8 $$\text{j}=\frac{\int_{\text{0}}^{\text{a}}\text{F}(\lambda )\varepsilon (\lambda )\lambda^{\text{4}}\text{d}\lambda }{\int_{\text{0}}^{\text{a}}\text{F}(\lambda )\text{d}\lambda }$$

In Eq. [7], K^2^ is the orientation factor related to the geometry of the donor and acceptor of dipoles and K2=2/3 for random orientation as in fluid solution, N is the average refractive index of medium in the wavelength range where spectral overlap is significant; F is the fluorescence quantum yield of the donor; J is the effect of the spectral overlap between the emission spectrum of the donor and the absorption spectrum of the acceptor, J could be calculated by Eq. [8], where, F(λ) is the corrected fluorescence intensity of the donor in the wavelength range of λ to λ+△λ; ε (λ) is the extinction coefficient of the acceptor at λ. FRET is an important technique to investigate a variety of biological phenomena including energy transfer processes.

**Figure 4:**
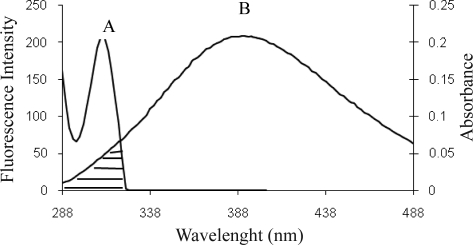
Spectral overlapping between the UV absorption spectrum of erlotinib hydrochloride (A) and the fluorescence emission of BSA (B).

The spectral overlap between UV/vis absorption spectrum of erlotinib hydrochloride (acceptor fluorophore) and the fluorescence emission spectrum of free BSA (donor fluorophore) is shown in figure 4. Since the fluorescence emission of protein was affected by the excitation light around 288 nm, the spectrum ranging from 288 to 488 nm was chosen to calculate the overlapping integral.

On the basis of equations 6-8, N= 1.36, and F= 0.15 (8) J was calculated to be 3.89×10-21 cm3 l mol-1,E=0.36, R=2 nm, and r=2.20 nm. The average distances between a donor and acceptor fluorophore is less than8 nm, and 0.5R<r<1.5R (14) presence of different concentrations of erlotinib suggesting that energy transfer occurs between BSA and erlotinib hydrochloride (8). Moreover, since r is higher than R, it suggests that erlotinib hydrochloride quench the intrinsic fluorescence of BSA by non- radiative energy transference and static quenching.

#### UV/Vis absorption spectroscopy

**Figure 5:**
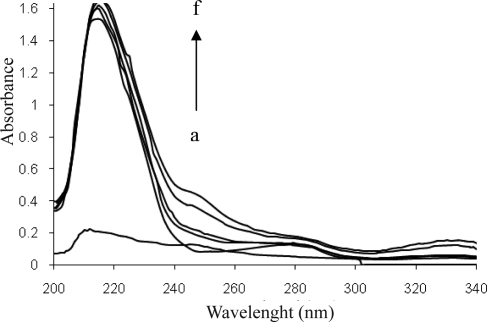
Absorption spectra of erlotinib hydrochloride, BSA, and erlotinib hydrochloride-BSA complex (a→f). BSA concentration was at 2×10^-6^ mol l^-1^. Erlotinib hydrochloride concentrations in E=0.36, R=2 nm, and r=2.20 nm. The average erlotinib hydrochloride -BSA complex were 2, 4, 6 and 8 ×10_−6_ mol l_−1_.

The absorption spectra of BSA in the presence and absence of erlotinib hydrochloride are illustrated in fig. 5. It was observed that the absorbance increased by increase in erlotinib hydrochloride concentration indicating the formation of a ground state complex. As dynamic quenching does not affect the absorption spectrum of quenching molecule and it only affects the exited states of quenching molecule, the observed changes in BSA absorbance in the hydrochloride could be indicative of the occurrence of static quenching interaction between erlotinib hydrochloride and BSA (15).

## CONCLUSION

The experimental results suggested that erlotinib hydrochloride can quench the intrinsic fluorescence of BSA through both dynamic and static quenching. The results also indicated that the hydrophobic interaction plays a major role in stabilization of the complex. The distance between BSA and erlotinib hydrochloride was obtained according to fluorescence resonance energy transfer. The changes of UV/vis absorption spectra were indicative of the formation of a ground state complex between erlotinib hydrochloride molecules and BSA.

## References

[1] Li Z, Xu M, Xing S, Ho W, Ishii T, Li Q, Fu X, Zhao Z (2007). Erlotinib Effectively Inhibits JAK2_V617F_ Activity and Polycythemia Vera Cell Growth. J. Biol. Chem.

[2] Thomas F, Rochaix P, White-Koning M, Hennebelle I, Sarini J, Benlyazid A, Malard L, Lefebvre J, Chatelut E, Delord J.P (2009). Population pharmacokinetics of erlotinib and its pharmacokinetic/pharmacodynamic relationships in head and neck squamous cell carcinoma. Eur J. Cancer.

[3] Olson R.E, Christ D.D (1996). Plasma Protein Binding of Drugs. Ann. Rep. Med. Chem.

[4] Papadopoulou A, Green R.J, Frazier R.A (2005). Interaction of Flavonoids with Bovine Serum Albumin: A Fluorescence Quenching Study. J. Agric. Food. Chem.

[5] Kanakis C.D, Tarantilis P.A, Polissiou M.G, Diamantoglou S, Tajmir-Riahi H.A (2006). Antioxidant flavonoids bind human serum albumin. J. Mol. Struct.

[6] Tang J.H, Luan F, Chen X.G (2006). Binding analysis of glycyrrhetinic acid to human serum albumin: Fluorescence spectroscopy, FTIR, and molecular modeling. Bioorg. Med. Chem.

[7] Chandregowda V, Rao G.V, Reddy G.C (2007). Convergent Approach for Commercial Synthesis of Gefitinib and Erlotinib. Org. Process Res. Dev.

[8] Hu Y.J, Liu Y, Zhang L.X (2005). Studies of interaction between colchicine and bovine serum albumin by fluorescence quenching method. J. Mol. Struct.

[9] Rasoulzadeh F, Najarpour H, Naseri A, Rashidi M.R (2009). Fluorescence quenching study of quercetin interaction with bovine milk xanthine oxidase. Spectrochim. Acta Part A.

[10] Wang Y, Zhang H, Zhang G, Tao W, Tang S (2007). Binding of brucine to human serum albumin. J. Mol. Struct.

[11] Li J, Li N, Wu Q, Wang Z, Ma J, Wang C, Zhang L (2007). Study on the interaction between clozapine and bovine serum albumin. J. Mol. Struct.

[12] Ross P.D, Subramanian S (1981). Thermodynamics of protein association reactions: forces contributing to stability. Biochemistry.

[13] Shohrati M, Rouini M.R, Mojtahedzadeh M, Firouzabadi M (2007). Evaluation of phenytoin pharmacokinetics in neurotrauma patients. DARU.

[14] Valeur B (2001). Molecular Fluorescence: Principles and Application.

[15] Hu Y.J, Liu Y, Pi Z.B, Qu S.S (2005). Interaction of cromolyn sodium with human serum albumin: A fluorescence quenching study. Bioorg. Med. Chem.

